# Comparative Analysis of Latex Transcriptome Reveals Putative Molecular Mechanisms Underlying Super Productivity of *Hevea* *brasiliensis*

**DOI:** 10.1371/journal.pone.0075307

**Published:** 2013-09-16

**Authors:** Chaorong Tang, Xiaohu Xiao, Heping Li, Yujie Fan, Jianghua Yang, Jiyan Qi, Huibo Li

**Affiliations:** 1 Rubber Research Institute, Chinese Academy of Tropical Agricultural Sciences, Danzhou, Hainan, China; 2 College of Agronomy, Hainan University, Haikou, Hainan, China; 3 Yunnan Tropical Crops Research Institute, Jinghong, Yunnan, China; Cinvestav, Mexico

## Abstract

Increasing demand for natural rubber prompts studies into the mechanisms governing the productivity of rubber tree ( *Hevea* *brasiliensis* ). It is very interesting to notice that a rubber tree of clone PR107 in Yunnan, China is reported to yield more than 20 times higher than the average rubber tree. This super-high-yielding (SHY) rubber tree (designated as SY107), produced 4.12 kg of latex (cytoplasm of rubber producing laticifers, containing about 30% of rubber) per tapping, more than 7-fold higher than that of the control. This rubber tree is therefore a good material to study how the rubber production is regulated at a molecular aspect. A comprehensive cDNA-AFLP transcript profiling was performed on the latex of SY107 and its average counterparts by using the 384 selective primer pairs for two restriction enzyme combinations (*Apo*I/*Mse*I and *Taq*I/*Mse*I). A total of 746 differentially expressed (DE) transcript-derived fragments (TDFs) were identified, of which the expression patterns of 453 TDFs were further confirmed by RT-PCR. These RT-PCR confirmed TDFs represented 352 non-redundant genes, of which 215 had known or partially known functions and were grouped into 10 functional categories. The top three largest categories were transcription and protein synthesis (representing 24.7% of the total genes), defense and stress (15.3%), and primary and secondary metabolism (14.0%). Detailed analysis of the DE-genes suggests notable characteristics of SHY phenotype in improved sucrose loading capability, rubber biosynthesis-preferred sugar utilization, enhanced general metabolism and timely stress alleviation. However, the SHY phenotype has little correlation with rubber-biosynthesis pathway genes.

## Introduction

Natural rubber (*cis*-1, 4-polyisoprene) is an important industrial raw material with wide applications. It has significant advantages, making it difficult to be replaced by synthetic alternatives in most applications [1]. *Hevea* *brasiliensis* (para rubber tree), a native tree of Brazil, is the sole commercial species cultivated for rubber production due to the high quality, high yield, easy harvesting and processing. Over 90% of natural rubber is produced in the Southeast Asia, particularly in Thailand, Indonesia and Malaysia. Increasing demand for rubber has driven a six-fold price increase since 2002, prompting studies into the mechanisms of high productivity, and a search for alternative sources of natural rubber [2]. Rubber biosynthesis occurs on the surface of a special type of organelle (rubber particle) in the cytoplasm (latex) of the rubber-producing laticifers or latex vessels [3].

Since the 1920s, great progress has been made in rubber breeding, mainly based on 33 seedlings of Wickham trees. In Malaysia, a series of rubber clones (varieties) recommended for plantation displayed a gradual increase in the mean annual yield, 1175 kg/ha for PilB84 (selected in the 1920s), 1425 kg/ha for RRIM501 (1928-1931), 2000 kg/ha for RRIM600 (1937-1941), and 2125 kg/ha for RRIM712 (1947-1958). However, the yields of the most productive clones currently available are still much inferior to the theoretical yield of rubber trees, which predicted 7,000-12,000 kg/ha/yr [4]. Conventional hand-pollination based rubber breeding is now confronted with a number of challenges for developing more productive rubber clones. These challenges include the long time needed for accurate selection (~30 yrs), the low female fertility that limits recombination, and the difficulty in efficiently utilizing new germplasms. Genetic transformation is a promising technique to overcome these barriers. Current rubber clones can be intentionally improved by transgenic engineering if molecular mechanisms underlying rubber productivity are well understood and the key genes involved are identified. The recent release of a draft Hevea genome [5] and other ongoing Hevea genome sequencing programs will be greatly beneficial to this purpose.

In *H* *. brasiliensis* , the general metabolic pathway leading to rubber biosynthesis (RB) is now clear [3], and all the genes involved have been identified, especially owing to the application of Illumina second generation sequencing technology in the latex and bark of Hevea tree [6-8]. Some of the RB genes have been cloned and further characterized, notably the *cis*-prenyltransferase (CPT) [2,10], rubber elongation factor (REF) [9,11], small rubber particle protein (SRPP) [12], and hydroxymethyglutaryl coenzyme A reductase (HMGR) [13]. However, the expression of the genes involved in other metabolic pathways within the latex cells can be essential in latex regeneration, especially in the case of ethylene-stimulated latex production [3]. For example, the expression of a sucrose transporter, *HbSUT3* (=*HbSUT1B*) [14,15] and two aquaporins [16,17] revealed to be significantly stimulated in the latex by ethylene treatment, and correlated with the effect of yield stimulation. In contrast, the expression of REF is little affected by ethylene treatment, suggesting REF is not a limiting factor in the case of ethylene-stimulated latex regeneration [3]. On the whole, the knowledge we have about the molecular regulation of rubber productivity is still scarce.

In a rubber plantation of Mengla County in Yunnan Province of China, a rubber tree of clone PR107 planted in 1963 produced 126 kg of dry rubber in 2002, over 20-fold higher than the average rubber trees [18]. We honored this tree as a super-high-yielding (SHY) PR107 tree, and designated it as SY107. To our knowledge, SY107 is the most productive rubber tree ever reported in the literature, and is a precious material for investigating the molecular regulation of rubber yield. The rubber trees presently exploited for rubber production are propagated by grafting axillary buds (scions) of elite clones onto unselected seedlings (rootstocks), and are simply named after the elite clones that provide the scions. Since rubber tree is a cross-pollinated crop, rootstock plants obtained by germinating open pollinated seeds harvested from the fields are highly heterozygous. This can lead to stock-scion interactions. Large intraclonal variations observed in growth and yield of bud-grafted clones of Hevea are attributed to the genetic heterogeneity of the rootstocks [19]. Therefore, the occasional optimum combination of scion and rootstock may account for the SHY performance of SY107. Other hypotheses include special micro-environments, or beneficial genetic mutations in the scion. Whatever the real reason is, realization of the ultimate SHY phenotype will depend largely on the regulation of gene expression in the laticifers. The effects of gene expression on biological responses have been reported both in plants [20] and in animals [21]. Therefore, to investigate the high-yielding molecular mechanisms of SY107, it is necessary to compare the latex transcriptome of SY107 with that of its average counterparts, and identify the differentially expressed (DE) genes. cDNA-amplified fragment length polymorphism (cDNA-AFLP) is a sensitive, reproducible, and high throughput technique for isolating DE- genes and is also a powerful tool in understanding the molecular mechanisms of a complex phenotype in non-model organisms [22-28]. A radioactive-labeling cDNA-AFLP analysis has been successfully applied to identify the genes that are differentially expressed in latex compared to leaf tissues [29]. Here, a silver-staining cDNA-AFLP procedure established for latex transcript profiling [30] was exploited to extensively compare the latex transcriptome of SY107 with that of the average PR107 trees. All the 384 selective primer pairs for two restriction enzyme pairs (*Apo*I/*Mse*I and *Taq*I/*Mse*I) were employed, and such analysis can theoretically cover more than 90% of the latex entire transcriptome [30].

## Results

### Latex yields and physiological parameters

Measured for three rounds of Etherl (an ethylene generator) stimulation (9 tappings in total), the mean latex yield of the SY107 tree was 4.12 kg per tapping, whereas it was only 0.32 kg and 0.83 kg for the two controls 107A and 107B, respectively. To have a preliminary knowledge about the physiological difference in the latex between SY107 and its average controls, four latex physiological parameters were determined. Table 1 shows the values of total solid content (TSC) and inorganic phosphorus (Pi) content were not significantly different between SY107 and the controls. TSC has dual effects on rubber productivity: limiting latex flow at a certain higher threshold but revealing an insufficient rubber synthesis activity at a certain lower threshold [31]. Pi participates in the regulation of glycolysis and in the formation of ATP, which is correlated with rubber production [32]. Sucrose content in SY107 was in a value of abundance range [33,34], but significantly lower than that in the control trees (Table 1), suggesting a more active sucrose catabolism in the laticifers of SY107. The thiol content of SY107 was significantly lower than that of the controls, but still maintained a value within the normal range [33,34]. The latex thiols play roles in maintaining the normal functions of the laticifers [35].

**Table 1:** Physiological parameters for the latex of super-high-yielding PR107 rubber tree and its average counterparts.

Physiological parameters	PR107 rubber trees	
	SY107	Control trees
Total solid content (%)	39.5 ± 0.8 a*	37.9 ± 1.3 a
Sucrose (mM)	20.95 ± 0.16 a	34.20 ± 0.12 b
Inorganic phosphorus (mM)	11.72 ± 0.32 a	12.14 ± 0.18 a
Thiols (mM)	0.596 ± 0.062 a	0.744 ± 0.021 b

* Different letters in the same row indicate statistical significance at *P*<0.05 while the same letter indicates no statistical significance.

### Differential expression of TDFs

**Figure 1:**
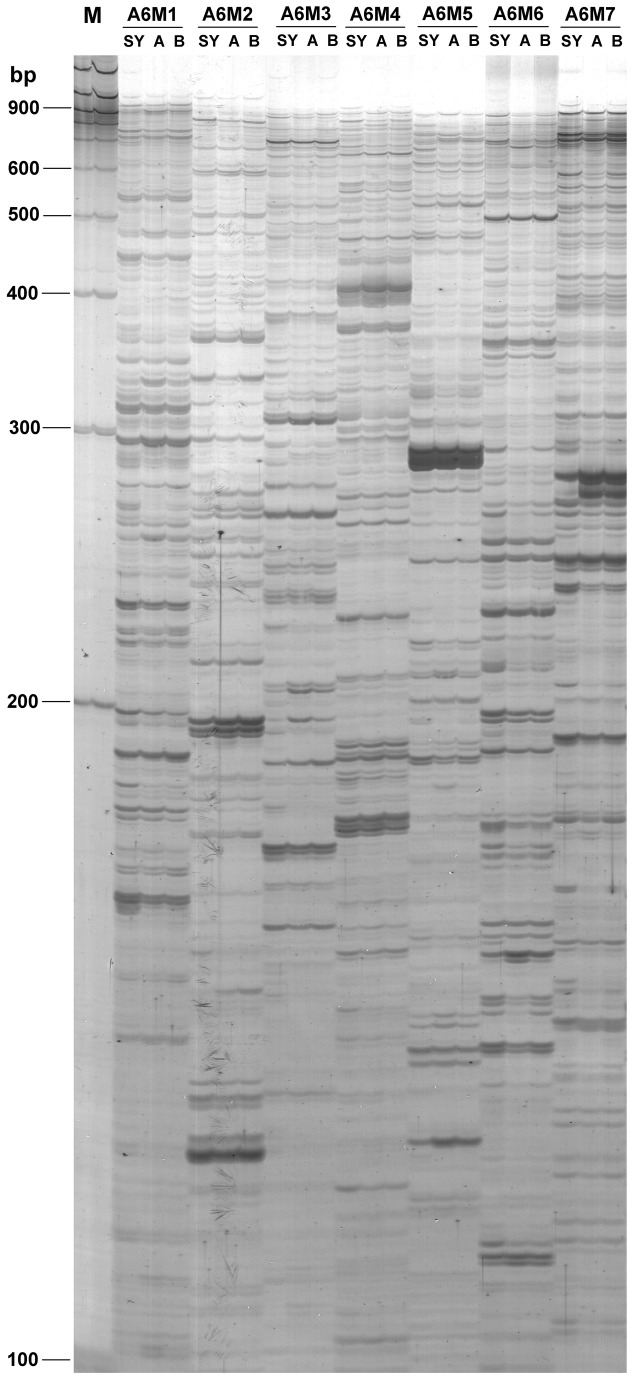
Example of a cDNA-AFLP fingerprint after silver staining. Seven selective primer combinations (A6M1-A6M7) are shown as an example. Lanes labeled SY, A and B were derived from latex RNA samples of SY107 and the two controls, 107A and 107B, respectively. Lanes labeled M indicates the 100-bp DNA ladder.

Most discernible bands appeared in the range of 150-700 bp, and beyond this range fewer bands were observed (Figure 1). For each primer combination, usually 80-90 transcript-derived fragments (TDFs) were visualized and a total of more than 35,000 TDFs were counted for all primer combinations. A total of 1038 differentially expressed (DE) TDFs were recovered and sequenced successfully, corresponding to about 3% of all visualized transcripts. After online BLAST analysis, 158 putative contaminated TDFs were discarded, which showed significant homology to the sequences of animals, fungi and bacteria. This left 880 TDFs for subsequent analysis.

All the TDFs in the format of FASTA were clustered using CAP3, and yielded 746 unique TDFs with average length of 306 bp, which included 98 contigs and 648 singletons. These DE-TDFs were BLASTN searched against the 48768 *Unigenes* assembled recently from the rubber leaf and latex transcriptomes [7]. According to the criterion of sharing ≥98% nucleotide identity as the same gene, out of the 746 DE-TDFs we isolated, 477 ones corresponded to 594 assembled *Unigenes* . The expression patterns for 60.7% (453) of the TDFs were further verified by semi-quantitative RT-PCR (sqPCR). The sqPCR-verified TDFs were annotated by similarly searching using the BLASTX program against the GenBank non-redundant public sequence database. To increase the rate of TDF annotation and reduce the redundancy of the acquired DE-TDFs, all sqPCR-verified TDFs were further clustered with the 20126 high-quality Sanger ESTs of SY107 latex [36], and further annotated according to the assembled contigs. The TDFs that showed above 98% sequence identity among their aligned portions (≥ 40 bp) with the same contig, and displayed similar expression patterns, were due to derive from the same mRNA transcript. In these cases, only the longest ones were characterized further. Finally, only 352 TDFs were subjected to further analysis, which may represent most of the non-redundant genes differentially expressed between the SY107 and its average controls (Tables 2 and 3).

**Table 2:** Annotation and functional classification of the DE-TDFs up-regulated in the latex of SY107 trees compared to its average counterparts.

TDF	Length(bp)	Blast searching				
		Genbank accession		Annotation (species)		E-value
**01 Primary and secondary metabolism**								
T15M1-1	517	XP_002513255		alcohol dehydrogenase, putative [*R. communis*]		2E-94
T2M12-2	253	XP_002515027		steroid dehydrogenase, putative [*R. communis*]		1E-18
T4M11-3	180	XP_002511334		copper amine oxidase, putative [*R. communis*]		6E-29
T7M5-2	283	AAN63056		dihydroflavonol reductase [P. tremuloides]		4E-32
T16M8-1	483	XP_002514996		ketoacyl-ACP Reductase (KAR) [*R. communis*]		1E-59
T13M4-3	80	XP_002525172		dolichyldiphosphatase, putative [*R. communis*]		2E-08
T14M16-1	532	XP_002511378		anthranilate phosphoribosyltransferase, putative [*R. communis*]		2E-87
T10M2-5	116	ABB89014		CXE carboxylesterase [ *A* *. arguta* ]		3E-09
T9M11-1	360	XP_002520220		UDP-glucosyltransferase, putative [*R. communis*]		1E-50
A5M8-2	337	XP_002524657		stachyose synthase precursor, putative [*R. communis*]		1E-54
A7M6-2	258	NP_180811		dienelactone hydrolase family protein [*A. thaliana*]		6E-32
T8M10-3	99	ACN66755		thiazole biosynthetic enzyme [*C. papaya*]		7E-12
A4M2-1	292	XP_002531475		mannose-6-phosphate isomerase, putative [*R. communis*]		2E-28
T13M2-6	186	BAH03299		GDP-D-mannose-3',5'-epimerase [*P. persica*]		1E-20
A1M5-3	209	XP_002528703		O-glycosyl hydrolase, putative [*R. communis*]		3E-32
T6M6-2	345	XP_002520975		trytophan synthase alpha subunit, putative [*R. communis*]		4E-49
A8M8-1	236	AAO92256		gamma-aminobutyrate transaminase subunit precursor isozyme 2 [S. lycopersicum]		8E-27
T15M3-4	141	XP_002518302		inorganic pyrophosphatase, putative [*R. communis*]		9E-09
T3M4-3	302	XP_002520803		biotin carboxyl carrier protein of acetyl-CoA carboxylase [*R. communis*]		2E-06
T2M8-1	492	ACN88681		VTC2-like protein [Malus x *domestica*]		8E-74
A6M1-2	167	XP_002513571		spermidine synthase 1, putative [*R. communis*]		3E-14
T6M1-1	248	XP_002526709		asparagine synthetase, putative [*R. communis*]		5E-31
T4M7-1	298	AAY85187		pyridoxal kinase [*G. max*]		6E-33
A5M10-2	390	XP_002518755		methylcrotonoyl-CoA carboxylase beta chain [*R. communis*]		1E-52
**02 Energy**								
T9M8-1	327	XP_002524666		Cytochrome c oxidase polypeptide Vc-2 [*R. communis*]		8E-19
T11M4-1	356	XP_002525025		phosphofructokinase, putative [*R. communis*]		7E-52
A1M9-2	119	XP_002516495		phosphofructokinase, putative [*R. communis*]		2E-13
T9M16-1	255	XP_002515391		chlorophyll a oxygenase, putative [*R. communis*]		4E-41
T9M11-2	181	XP_002529227		cytochrome P450, putative [*R. communis*]		4E-22
T4M12-3	188	XP_002521002		cytochrome P450, putative [*R. communis*]		1E-23
**03 Cell, structure, growth and division**								
T13M2-1	508	XP_002520329		cyclin A, putative [*R. communis*]		2E-32
T2M8-2	471	ABA96359		Nse1 non-SMC component of SMC5-6 complex family protein [*O. sativa* (japonica cultivar)]		3E-45
T8M10-1	333	AAK84479		putative auxin growth promotor protein [S. lycopersicum]		3E-49
A2M2-1	298	XP_002525631		villin 1-4, putative [*R. communis*]		3E-42
T7M8-3	186	XP_002512241		DNA-damage-repair/toleration protein DRT102 [*R. communis*]		7E-20
T16M5-3	173	XP_002520219		gibberellin-regulated protein 3 precursor [*R. communis*]		2E-23
T12M5-3	258	AAR07598		fiber protein Fb19 [G. barbadense]		1E-29
T12M12-2	364	AAC33277		cotton fiber expressed protein 2 [*G. hirsutum*]		7E-17
T9M11-3	129	XP_002530862		glucan endo-1,3-beta-glucosidase precursor [*R. communis*]		2E-15
T16M10-2	233	O65812		profilin [ *H* *. brasiliensis* ]		1E-37
A4M6-1	385	BAC78827		caffeic acid O-methyltransferase [ *R* *. chinensis* var. spontanea]		4E-50
T15M10-2	176	XP_002525818		o-methyltransferase, putative [*R. communis*]		1E-12
T2M15-1	510	XP_002511717		cinnamoyl-CoA reductase, putative [*R. communis*]		1E-59
T5M4-3	308	XP_002297654		10-formyltetrahydrofolate synthetase [ *P* *. trichocarpa* ]		9E-39
T13M10-3	185	XP_002522819		histone h2a, putative [*R. communis*]		2E-26
**04 Transcription and protein synthesis**								
T4M10-3	186	NP_566412		KH domain-containing protein / zinc finger (CCCH type) family protein [*A. thaliana*]		3E-17
T8M5-1	337	ACT78956		zinc finger protein [G. arboreum]		4E-16
A2M10-1	257	XP_002532252		zinc finger protein, putative [*R. communis*]		2E-20
T14M5-1	288	NP_568590		zinc finger (C3HC4-type RING finger) family protein [*A. thaliana*]		2E-14
A8M2-2	189	ABH02845		MYB transcription factor MYB93 [*G. max*]		1E-18
T7M16-4	125	BAD28879		myb family transcription factor-like [*O. sativaJaponica* Group]		2E-13
T15M3-2	358	XP_002308691		AP2/ERF domain-containing transcription factor [ *P* *. trichocarpa* ]		2E-31
T6M2-1	358	XP_002515379		transcription factor, putative [*R. communis*]		1E-26
T14M16-4	176	XP_002531463		homeobox protein, putative [*R. communis*]		1E-13
T16M11-1	303	ACL51015		squamosa promoter-binding protein [ *C* *. trifoliata* ]		8E-24
T7M10-3	166	XP_002527359		transcription initiation factor iia (tfiia), gamma chain [*R. communis*]		1E-20
T4M12-2	232	ABD32320		DNA-directed RNA polymerase, subunit C11/M/9 [*M. truncatula*]		3E-34
T6M6-1	416	XP_002518700		DNA-directed RNA polymerase II subunit, putative [*R. communis*]		7E-67
T4M2-1	314	XP_002529501		Heterogeneous nuclear ribonucleoprotein A1, putative [*Ricinus communis*]		7E-23
T9M10-1	328	NP_174334		CPSF30; RNA binding/calmodulin binding/endonuclease[*Arabidopsis thaliana*]		1E-45
T3M2-1	394	XP_002530100		U4/U6 small nuclear ribonucleoprotein Prp4, putative [*R. communis*]		4E-64
T11M10-1	342	XP_002528794		nonsense-mediated mRNA decay protein, putative [*R. communis*]		2E-26
T4M6-1	212	BAF01501		Sm-like protein [*A. thaliana*]		4E-12
A2M2-2	161	XP_002513402		yth domain-containing protein, putative [*R. communis*]		3E-10
A3M2-1	115	XP_002513402		yth domain-containing protein, putative [*R. communis*]		2E-07
T11M10-2	335	NP_567592		GCN5-related N-acetyltransferase (GNAT) family protein [*A. thaliana*]		1E-10
A1M7-1	340	XP_002512829		DNA binding protein, putative [*R. communis*]		6E-14
T8M9-1	288	XP_002525167		DNA-binding protein S1FA, putative [*R. communis*]		5E-07
T3M16-2	191	XP_002511085		DNA binding protein, putative [*R. communis*]		2E-15
T5M2-2	432	XP_002515833		nucleic acid binding protein, putative [*R. communis*]		7E-70
T6M12-1	361	XP_002532850		nucleic acid binding protein, putative [*R. communis*]		6E-50
T5M7-2	217	XP_002512575		nucleic acid binding protein, putative [*R. communis*]		8E-27
T14M10-1	230	XP_002512132		eukaryotic translation initiation factor 5a, putative [*R. communis*]		1E-37
T15M1-3	297	ACG38773		eukaryotic translation initiation factor 2 gamma subunit [*Z. mays*]		2E-18
T9M4-2	309	XP_002511200		elongation factor 1-beta, putative [*R. communis*]		1E-24
T5M4-1	307	XP_002509506		ribosomal protein L27, putative [*R. communis*]		1E-49
T15M8-2	291	AAR83866		40S ribosomal protein S17 [*C. annuum*]		4E-42
T10M9-1	297	XP_002521598		40S ribosomal protein S12, putative [*R. communis*]		1E-26
T11M13-1	514	XP_002526494		40S ribosomal protein S11, putative [*R. communis*]		1E-74
T15M1-4	185	ACM90156		40S ribosomal protein S15-like protein [J. curcas]		9E-23
T3M14-2	204	XP_002519689		40S ribosomal protein S14, putative [*R. communis*]		3E-22
T10M1-1	352	XP_002511695		40S ribosomal protein S13, putative [*R. communis*]		3E-19
T15M3-1	629	XP_002530136		60S ribosomal protein L18a, plant, putative [*R. communis*]		2E-24
T11M8-3	230	XP_002530279		60S ribosomal protein L12, putative [*R. communis*]		3E-34
A6M2-1	329	ACJ02347		60S ribosomal protein L17 [ *V* *. fordii* ]		2E-36
T12M13-1	258	NP_001151284		60S ribosomal protein L5-1 [*Z. mays*]		2E-38
T15M7-1	158	XP_002526873		60S acidic ribosomal protein P0, putative [*R. communis*]		2E-21
T16M3-1	355	XP_002527874		60S ribosomal protein L35a, putative [*R. communis*]		9E-36
A2M4-1	332	XP_002515180		prolyl-tRNA synthetase, putative [*R. communis*]		4E-58
**05 Protein destination and storage**								
T2M7-1	326	XP_002533817		ubiquitin-conjugating enzyme E2 j2, putative [*R. communis*]		9E-20
T3M3-2	140	XP_002265672		ubiquitin-protein ligase [*V. vinifera*]		4E-16
T11M2-1	470	NP_565522		ubiquitin-protein ligase [*A. thaliana*]		8E-43
T16M2-1	169	XP_002517911		ubiquitin conjugating enzyme, putative [*R. communis*]		3E-15
A8M12-1	402	ACV49920		ubiquitin-conjugating enzyme variant [*C. sinensis*]		2E-26
A4M12-3	328	XP_002513950		ubiquitin-protein ligase, putative [*R. communis*]		3E-28
A5M7-1	493	XP_002521028		ring finger protein, putative [*R. communis*]		3E-38
T13M2-3	411	XP_002282071		similar to ubiquitin fusion protein [*V. vinifera*]		2E-54
T12M7-1	224	NP_565253		RHA2B (RING-H2 FINGER PROTEIN 2B) [*A. thaliana*]		2E-21
T12M2-2	134	XP_002308717		f-box family protein [ *P* *. trichocarpa* ]		2E-13
A5M11-2	160	ACB87912		F-box-containing protein 2 [Malus x *domestica*]		1E-10
A7M12-1	245	NP_198752		EDL2 (EID1-like F-box protein 2) [*A. thaliana*]		1E-36
A4M10-1	479	XP_002530834		26S proteasome non-atpase regulatory subunit [*R. communis*]		2E-49
T1M12-1	440	XP_002525692		BTB and MATH domain-containing protein [*R. communis*]		9E-63
T9M9-1	234	XP_002511285		protein-l-isoaspartate O-methyltransferase, putative [*R. communis*]		2E-34
T7M14-2	219	NP_171696		peptidyl-prolyl cis-trans isomerase cyclophilin-type family protein [*A. thaliana*]		9E-28
T14M7-2	566	XP_002285871		peptidase isoform 1 [*V. vinifera*]		7E-99
T4M10-1	435	XP_002532339		protein translocase, putative [*R. communis*]		2E-41
**06 Transporters and intracellular traffic**								
A1M5-1	700	ACV66986		plasma membrane aquaporin 2 [ *H* *. brasiliensis* ]		1E-54
A7M5-1	220	XP_002516442		zinc/iron transporter, putative [*R. communis*]		3E-21
T4M6-2	205	XP_002522484		vesicle transport V-snare protein vti1a, putative [*R. communis*]		1E-20
T3M10-2	232	EF067334		sucrose transporter 3 [ *Hevea* *brasiliensis* ]		1E-70
A5M11-1	272	XP_002336439		magnesium transporter [ *P* *. trichocarpa* ]		2E-35
T11M8-2	312	ACX37450		plasma membrane intrinsic protein (PIP2;1) [ *H* *. brasiliensis* ]		7E-56
T12M2-1	265	BAB90396		ADP-ribosylation factor [*O. sativaJaponica* Group]		1E-11
T13M10-1	326	XP_002516060		mitochondrial carnitine/acylcarnitine carrier protein[*R. communis*]		6E-08
A3M15-1	102	XP_002523748		vacuolar protein sorting vps41, putative [*R. communis*]		6E-11
A4M11-1	298	BAF01013		putative Rab5-interacting protein - like [*A. thaliana*]		8E-32
**07 Signal transduction**								
A5M6-2	372	XP_002526147		brassinosteroid LRR receptor kinase precursor [*R. communis*]		3E-33
T7M1-2	298	XP_002529785		dimethylaniline monooxygenase, putative [*R. communis*]		2E-28
T6M11-1	264	XP_002510007		receptor protein kinase CLAVATA1 precursor [*R. communis*]		3E-37
T11M1-2	320	XP_002510650		receptor protein kinase, putative [*R. communis*]		2E-44
T15M13-1	81	AAP88291		protein kinase [*C. sativus*]		2E-06
A6M5-1	633	XP_002527541		histidine kinase 1, 2, 3 plant, putative [*R. communis*]		2E-70
A6M9-3	94	AAK44123		putative casein kinase II, alpha chain 2 CK II [*A. thaliana*]		2E-09
T7M5-1	451	XP_002510808		steroid binding protein, putative [*R. communis*]		2E-28
T3M9-1	361	AAC62626		rac GTPase activating protein 3 [ *L* *. japonicus* ]		3E-10
T16M7-4	185	XP_002529557		receptor for activated protein kinase C [*R. communis*]		4E-26
T3M7-1	346	XP_002513509		nucleolar GTP-binding protein, putative [*R. communis*]		4E-60
T7M7-2	211	XP_002513080		annexin, putative [*R. communis*]		1E-26
T15M8-1	383	BAD88034		adenylyl cyclase-like [*O. sativaJaponica* Group]		3E-10
T8M10-4	99	XP_002521342		developmentally regulated GTP-binding protein [*R. communis*]		4E-11
A3M1-2	89	XP_002524007		phospholipase C 3 precursor, putative [*R. communis*]		6E-06
**08 Defense and stress**								
T4M11-1	194	XP_002530396		heat-shock protein, putative [*R. communis*]		1E-19
T15M6-1	507	XP_002531089		chaperone protein DNAj, putative [*R. communis*]		9E-58
T2M6-3	256	XP_002531285		wound-induced protein WIN1 precursor [*R. communis*]		3E-11
T10M15-3	236	CAA11041		latex allergen [ *H* *. brasiliensis* ]		1E-27
T7M9-1	223	ACI04518		translationally controlled tumor protein [ *H* *. brasiliensis* ]		6E-22
T12M1-1	443	ABB13620		USP-like protein [A. sinicus]		1E-36
T8M3-3	286	ABS12334		dehydrin [ *P* *. tremula* var. *davidiana* ]		5E-19
T14M4-1	334	XP_002318626		disease resistance protein [ *P* *. trichocarpa* ]		4E-10
T10M9-2	221	XP_002515414		glutathione peroxidase, putative [*R. communis*]		7E-36
T7M9-2	152	XP_002303583		glutathione peroxidase [ *P* *. trichocarpa* ]		2E-06
T1M16-4	132	XP_002527520		trehalose-6-phosphate synthase, putative [*R. communis*]		1E-08
T11M3-2	192	XP_002527520		trehalose-6-phosphate synthase, putative [*R. communis*]		3E-27
**09 Rubber biosynthesis**								
**10 Unclear classification**								
A3M12-2	513	NP_192096		tolB protein-related [*A. thaliana*]		4E-58
T1M12-3	104	XP_002522792		neigor of COX4, putative [*R. communis*]		5E-05
T4M8-1	503	XP_002524557		XPA-binding protein, putative [*R. communis*]		2E-60
T5M2-3	328	XP_002528475		leucoanthocyanidin dioxygenase, putative [*R. communis*]		4E-09
T5M8-3	215	XP_002515000		ankyrin repeat-containing protein, putative [*R. communis*]		4E-26
T13M7-2	349	XP_002517559		hydrolase, putative [*R. communis*]		1E-52
T6M6-3	282	XP_002509832		protein binding protein, putative [*R. communis*]		2E-36
T10M3-1	353	XP_002533745		catalytic, putative [*R. communis*]		1E-52
T10M1-2	309	XP_002511079		catalytic, putative [*R. communis*]		8E-50
T9M4-1	349	XP_002510571		ATP binding protein, putative [*R. communis*]		2E-12
T11M13-4	151	NP_973832		oxidoreductase NAD-binding domain-containing [*A. thaliana*]		2E-17
A5M12-4	116	XP_002518115		PMP, putative [*R. communis*]		2E-07
T3M6-2	443	XP_002510059		cw7 protein, putative [*R. communis*]		4E-59
T7M14-1	420	NP_564367		integral membrane HRF1 family protein [*A. thaliana*]		4E-50
**11 Unclassified**								
	41 DE-TDFs in total						
**12 No hit**								
	48 DE-TDFs in total						

**Table 3:** Annotation and functional classification of the DE-TDFs down-regulated in the latex of SY107 trees compared to its average counterparts.

TDF	Length (bp)	Blast searching			
		Genbank accession	Annotation (species)		E-value
**01 Primary and secondary metabolism**					
T1M1-3	191	XP_002514215	ceramide glucosyltransferase, putative [*R. communis*]		8E-16
T3M16-3	74	XP_002519980	ethanolamine-phosphate cytidylyltransferase, putative [*R. communis*]		6E-05
T14M13-3	167	XP_002527433	phosphatidylcholine: diacylglycerol acyltransferase [*R. communis*]		3E-19
T9M16-2	206	XP_002313788	phenylcoumaran benzylic ether reductase 3 [ *P* *. trichocarpa* ]		6E-26
A6M6-1	641	XP_002518196	beta-amylase, putative [*R. communis*]		1E-107
T14M4-2	256	XP_002513415	proteasome-activating nucleotidase, putative [*R. communis*]		6E-24
**02 Energy**					
T13M13-1	452	XP_002516221	adrenodoxin, putative [*R. communis*]		4E-36
T16M4-2	301	XP_002517532	D-glycerate 3-kinase, putative [*R. communis*]		3E-39
**03 Cell structure, growth and division**					
T3M1-1	313	XP_002517787	Angio-associated migratory cell protein, putative [*R. communis*]		3E-13
A7M4-1	206	XP_002526324	DNA gyrase subunit A [*R. communis*]		2E-16
**04 Transcription and protein synthesis**					
A7M9-2	183	XP_002514762	transcription factor, putative [*R. communis*]		2E-17
T16M13-1	577	AAD26942	zinc-finger protein 1 [ *D* *. glomerata* ]		2E-26
A1M9-3	98	ABF95225	zinc finger family protein [*O. sativa* (japonica cultivar-group)]		2E-11
T9M13-2	245	NP_001150575	transcription elongation factor SPT4 [*Z. mays*]		2E-29
T6M11-3	160	XP_002530669	RNA splicing protein mrs2, putative [*R. communis*]		9E-12
T9M13-3	152	XP_002515442	ccr4-associated factor, putative [*R. communis*]		2E-17
A1M13-1	466	XP_002520438	RNA binding motif protein, putative [*R. communis*]		4E-29
T5M16-3	79	XP_002523184	GTP-dependent nucleic acid-binding protein engD [*R. communis*]		3E-06
A1M15-1	415	ACS96446	60S ribosomal protein L18a [J. curcas]		5E-73
**05 Protein destination and storage**					
T7M4-4	196	XP_002512947	ubiquitin-protein ligase, putative [*R. communis*]		6E-21
A2M9-1	210	Q43207	peptidylprolyl isomerase [*T. aestivum*]		2E-28
A4M15-1	275	XP_002533551	ubiquitin ligase protein cop1, putative [*R. communis*]		3E-31
A8M12-2	238	XP_002526029	groes chaperonin, putative [*R. communis*]		1E-16
**06 Transporters and intracellular traffic**					
T6 M15-2	324	XP_002529745	sugar transporter, putative [*R. communis*]		9E-25
A8M2-1	193	ABJ51934	sucrose transporter 2A [ *H* *. brasiliensis* ]		5E-05
T13 M1-2	390	ABK60189	sucrose transporter 5 [ *H* *. brasiliensis* ]		1E-62
T3M5-1	307	NP_001150271	ras-related protein Rab-6A [*Z. mays*]		7E-47
A3M14-1	238	XP_002527996	cation transport protein chaC [*R. communis*]		2E-32
T7M3-3	349	XP_002532663	grave disease carrier protein, putative [*R. communis*]		4E-34
**07 Signal transduction**					
A3M10-1	772	XP_002527010	protein phosphatase, putative [*R. communis*]		1E-126
T10M4-3	152	BAA94510	protein kinase 2 [ *P* *. nigra* ]		6E-19
A5M2-1	411	NP_173259	calcium-binding protein, putative [*A. thaliana*]		2E-43
T6M16-1	303	NP_567979	cyclase family protein [*A. thaliana*]		7E-28
**08 Defense and stress**					
A6M7-1	246	XP_002520482	heat-shock protein, putative [*R. communis*]		5E-37
A4M1-1	312	XP_002529170	heat-shock protein, putative [*R. communis*]		7E-12
A4M1-2	120	XP_002520482	heat-shock protein, putative [*R. communis*]		2E-14
T2M11-3	216	XP_002520464	heat-shock protein, putative [*R. communis*]		2E-29
T3M10-1	218	XP_002516106	heat-shock protein, putative [*R. communis*]		9E-26
T3M15-2	360	XP_002532054	heat shock protein, putative [*R. communis*]		1E-37
T2M3-2	123	XP_002532054	heat shock protein, putative [*R. communis*]		4E-05
T6M1-2	179	XP_002520481	heat-shock protein, putative [*R. communis*]		1E-23
A5M9-2	327	XP_002526950	heat-shock protein, putative [*R. communis*]		3E-29
T7M11-1	317	XP_002520481	heat-shock protein, putative [*R. communis*]		6E-46
T8M1-1	387	XP_002530396	heat-shock protein, putative [*R. communis*]		3E-28
A5M15-1	538	XP_002526012	heat shock protein binding protein, putative [*R. communis*]	6E-27
A7M3-1	227	XP_002516783	heat shock protein 70kD, putative [*R. communis*]	5E-32
A8M6-2	281	CAB72128	heat shock protein 70 [*C. sativus*]	1E-36
T3M6-3	175	CAI94864.2	heat shock protein 101 [ *T* *. turgidum* subsp. *durum* ]	7E-25	
T2M3-1	353	XP_002517628	small heat-shock protein, putative [*R. communis*]	2E-22	
A4M11-3	262	CAA12387	Hsp20.1 protein [ *S* *. peruvianum* ]	2E-18	
T2M11-1	431	AAD41409	cytosolic class II low molecular weight heat shock protein [ *P* *. dulcis* ]	4E-49	
T9M5-2	274	ABW89469	low molecular weight heat shock protein [*G. hirsutum*]	1E-25	
T6M16-2	232	XP_002530362	Hsp90 co-chaperone AHA1, putative [*R. communis*]	9E-23	
A4M5-1	489	AAV36519	phosphosulfolactate synthase-related protein [S. lycopersicum]	2E-43	
**09 Rubber biosynthesis**					
A2M8-1	217	BAF98277	acetyl-CoA C-acetyltransferase [ *H* *. brasiliensis* ]	4E-08	
**10 Unclear classification**					
A7M8-2	231	CAP16621	polyprotein [*M. acuminata* subsp. *malaccensis* ]	2E-33	
T2M5-1	351	XP_002511089	nucleotide binding protein, putative [*R. communis*]	1E-55	
**11 Unclassified**					
	10 DE-TDFs in total
**12 No hit**					
	38 DE-TDFs in total

### qPCR validation of differential expression

**Figure 2:**
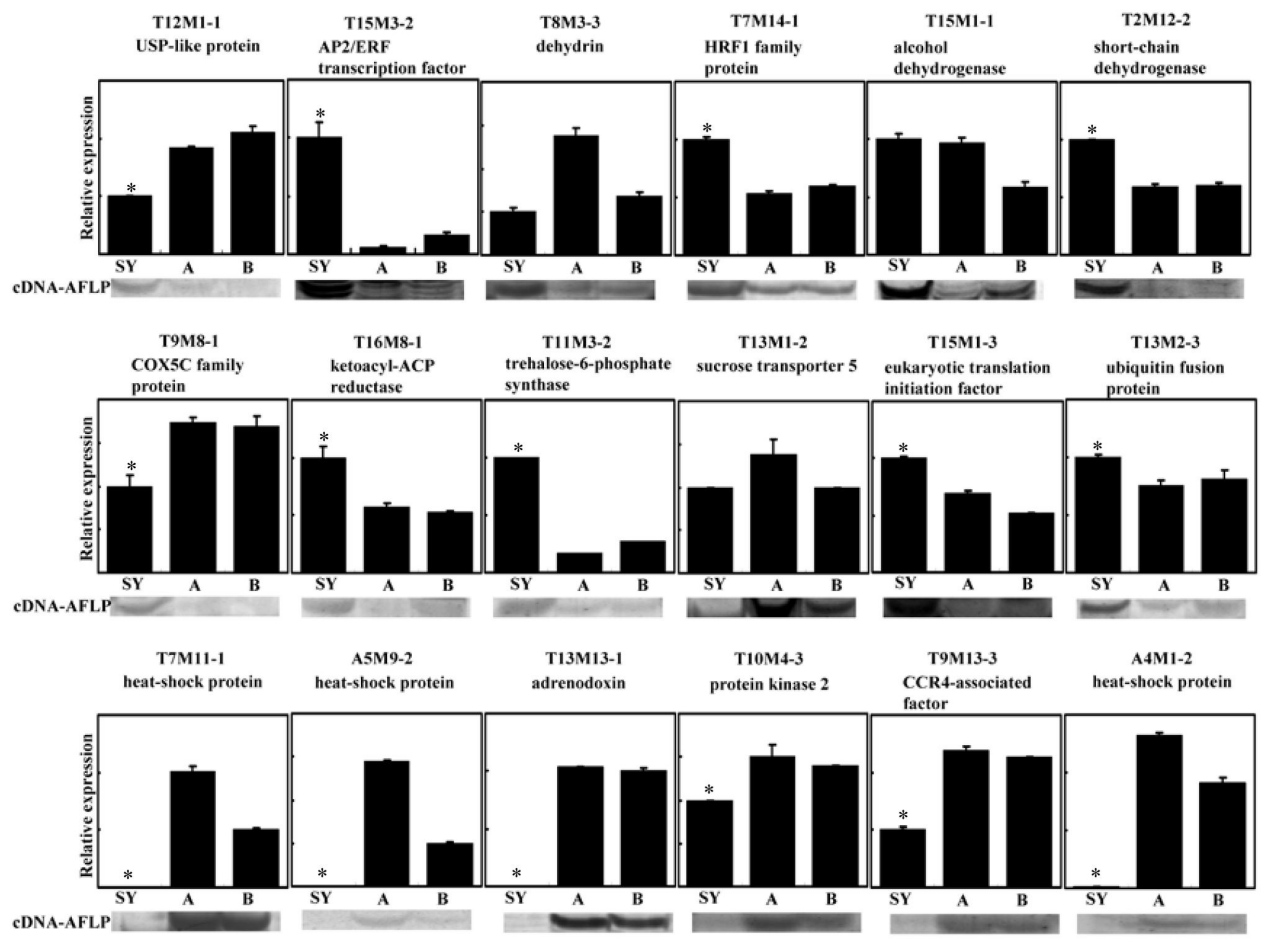
Comparison of expression patterns of 18 TDFs by both cDNA-AFLP and qRT-PCR. Templates derived from SY107 (SY) and the two controls, 107A (A) and 107B (B), respectively. The values of relative expression are presented as the mean ± SE of three biological replicates. An asterisk indicates the values are significantly different (*t* test, P < 0.05) between SY107 and the two controls.

To further validate the cDNA-AFLP expression patterns, 18 TDFs were selected for qPCR analysis. As shown in Figure 2, 13 of the 18 TDFs examined revealed the same expression profiles as in cDNA-AFLP analysis. However, the expression of the genes encoding an USP-like protein (T12M1-1), a dehydrin (T8M3-3) and a COX5C family protein (T9M8-1) showed different patterns. They were down-regulated in qPCR analysis, but up-regulated in cDNA-AFLP analysis (Figure 2). In addition, in cDNA-AFLP analysis an alcohol dehydrogenase gene (T15M1-1) was strikingly up-regulated especially with comparison to 107A, whereas in qPCR analysis, this gene displayed a similar expression between SY107 and 107A (Figure 2). The discrepancies between the results of qPCR and cDNA-AFLP analysis are probably due to gene family complexity or the ways to normalize the template amount. In cDNA-AFLP analysis, equal aliquots of total RNA were used for different samples at the beginning, or equal double-stranded cDNAs used at a later stage [28,30,37]. In qPCR analysis, an internal control gene, UBC2 in this study, was exploited to normalize the differences of the cDNA templates used. Nevertheless, the general approach used in this study proved reliable in identifying the genes differentially expressed between the SY107 and the average trees: 72.2% of the cDNA-AFLP patterns were confirmed by qPCR analysis (Figure 2).

### Functional classification of DE-genes

Of the 352 non-redundant DE-genes, 215 (61.1%) showed significant homology to genes with known or partially known functions. Fifty-one (14.5%) were homologous to predicted proteins or proteins with unknown functions, and were categorized as ‘unclassified’ (Tables 2 and 3). The final 86 (24.4%) didn’t show significant matches to available public sequences.

**Figure 3:**
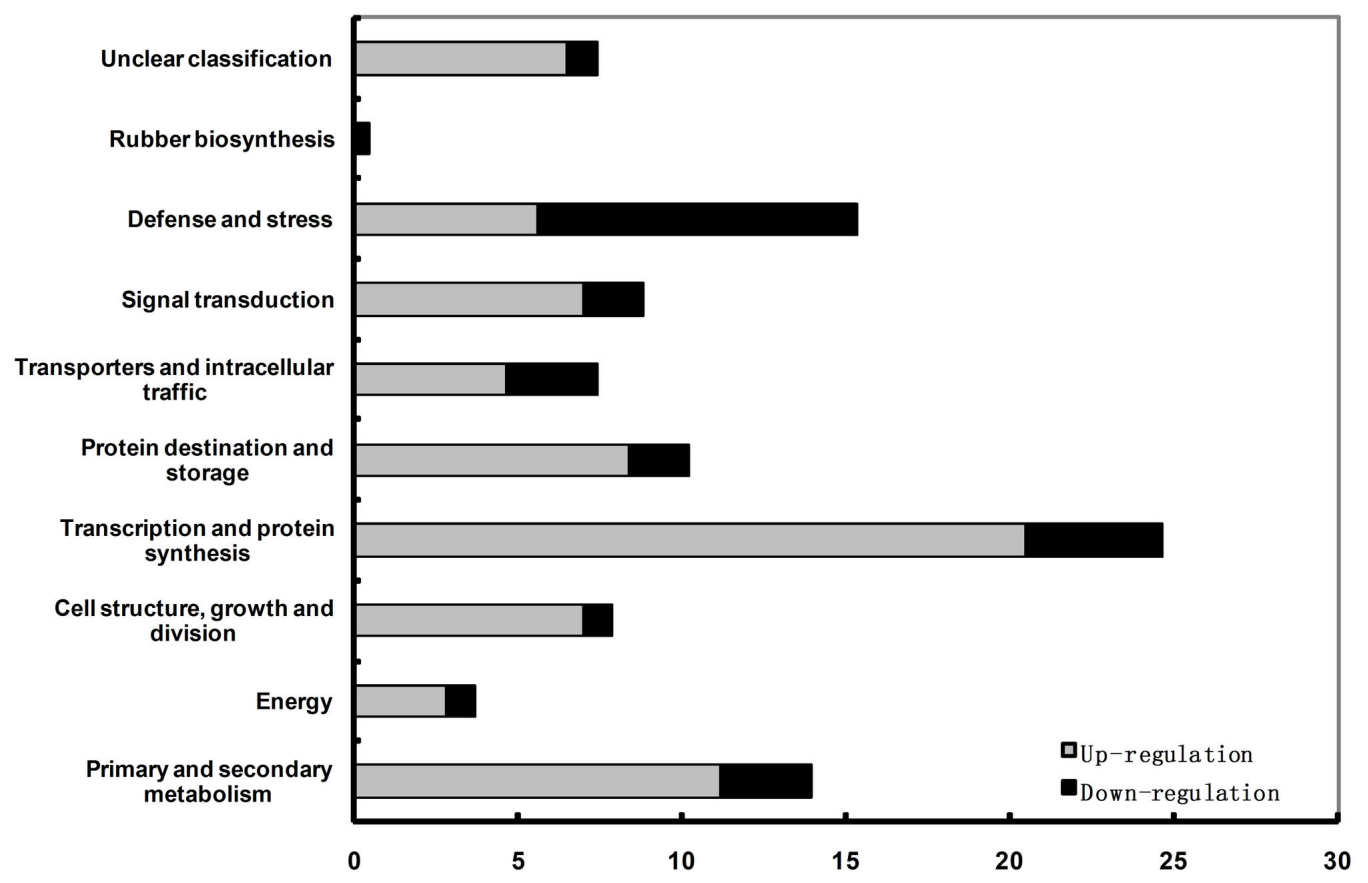
Functional classification of non-redundant genes with known or putative function differentially expressed between SY107 and its control trees displayed by cDNA-AFLP. The percentages of up-regulated (in light grey) and down-regulated (in black) transcripts within each functional category were shown, which was primarily based on the data displayed in tables 2 and 3.

The 215 DE-genes with known or partially known functions were grouped into ten functional categories (Figure 3; GenBank_Accn: JZ479023-JZ479237). Transcription and protein synthesis, the largest category, represented 24.7% of the annotated sequences. The genes involved in defense and stress were the second largest category (15.3%), and followed by the category of primary and secondary metabolism (14.0%). These three categories add up to 54.0% of the total sequences, revealing the major biological differences that occur in the laticifers between SY107 and the average trees. Protein destination and storage comprises 10.2% of the total sequences, taking up the fourth place. Other categories include signal transduction (8.8%), cell structure, growth and division (7.9%), unclear classification (7.4%), transporters and intracellular traffic (7.4%), energy (3.7%), and RB (0.5%). The category of unclear classification includes the genes with only partially known functions, and unable to be assigned into any definite functional category. Most of the DE-genes were up-regulated (73.5%) in SY107 compared with the average trees. There are two exception categories, viz., defense and stress, and rubber biosynthesis. For the category of defense and stress, 63.6% of the DE-genes were down-regulated. In the category of rubber biosynthesis, only one DE-gene was identified and revealed to be down-regulated.

### Predicted function of DE-genes

In total, 30 DE-genes were in the category of primary and secondary metabolism (Tables 2 and 3), of which 24 were up-regulated. Some of the up-regulated genes were involved in basic metabolic functions, such as carbohydrate metabolism, amino acid metabolism, fatty acid synthesis, lipid degradation, vitamin metabolism, and alcohol fermentation (Table 2). The other up-regulated genes played roles in secondary metabolism, such as the metabolism of amines and steroids, chlorocatechol degradation, and anthocyanin synthesis. The down-regulated DE-genes were involved in lipid metabolism, phenylpropanoid synthesis, nucleotide hydrolysis, and polysaccharide breakdown.

Of the eight DE-genes in the energy category, six were up-regulated and involved in glycolysis, electron transport chain and chlorophyll b synthesis (Table 2). The two down-regulated genes were in the photorespiratory C2 cycle and electron transfer (Table 3). Fifteen up-regulated and two down-regulated DE-genes were grouped in cell structure, growth and division (Tables 2 and 3). Of the up-regulated genes, three were involved in DNA synthesis and repair, and cell cycle; the others were implicated in cytoskeleton, cell wall structure modification, cell growth and division. The two down-regulated genes were predicted in DNA replication and cell growth.

The largest functional category, i.e. transcription and protein synthesis included 53 DE-genes, the majority (44) of which was up-regulated (Tables 2 and 3). Fifteen genes encoded various types of transcription factors (TFs), including six zinc-finger containing TFs, two MYB or MYB-related family TFs, one HB family TF, one SBP family TF, one AP2/ERF family TF, two general TFs, and two unknown type of TFs. Through regulating the expression of a variety of target genes, these types of TFs are implicated in almost all known biological processes in higher plants, such as cell proliferation and differentiation, growth and development, and various responses to environmental stimuli [38-41]. Nineteen genes were directly involved in protein synthesis, including fourteen ribosomal proteins, four translation factors, and one aminoacyl tRNA synthase. Twelve genes were involved in mRNA synthesis, processing and stability control. The remaining seven genes encoded nucleic acid (DNA, RNA or both)-binding proteins, being putative regulators of transcription or translation.

The category of protein destination and storage included 22 DE-genes, only four of which were down-regulated (Tables 2 and 3). Most genes in this category encoded proteins involved in unbiquitin-mediated protein degradation [42], such as ubiquitin-conjugating enzymes, ubiquitin-protein ligases, F-box proteins, ring-finger proteins, and 26S proteasome and peptidase. The other genes were involved in protein folding, protein damage repair, and protein targeting. In the category of transporters and intracellular traffic, 16 DE-genes were included, of which six were down-regulated (Tables 2 and 3). Of the up-regulated genes, two aquaporins, one sucrose transporter, four vesicle trafficking proteins, two ions transporters, and one carnitine carrier protein were identified. The down-regulated genes included two sucrose transporters, one putative sugar transporter, one Rab protein, one cation transport protein, and one grave disease carrier protein.

Nineteen genes were assigned in the category of signal transduction, most (15) of which were up-regulated (Tables 2 and 3). Some important signaling components were included, such as protein kinases, phosphatases, receptors, and G proteins, which play roles in a number of well-known signaling pathways [43-47]. Thirty-three DE-genes were grouped in the category of defense and stress, consisting of the second largest category. It is the only category that has a larger quantity of down-regulated genes (21) than up-regulated (12) (Tables 2 and 3). The 12 up-regulated genes consisted of a variety of stress and defense-related genes (Table 2), such as molecular chaperons, detoxification enzymes, osmoprotectant-biosynthesis related proteins, disease resistance proteins and so on. Interestingly, in this category, nearly all down-regulated genes belonged to the family of heat shock proteins (Table 3). The exception one was a phosphosulfolactate synthase-related protein (A4M5-1), which also reveals to be a novel heat shock protein [48]. Only one DE-gene was identified in the category of rubber biosynthesis, and it was a down-regulated acetyl-CoA C-acetyltransferase (Tables 2 and 3).

## Discussion

Rubber plantations in China are located at 18-24 ° N, entirely beyond the traditional rubber-growing tract in the world, and are often subjected to serious damages caused by typhoons and cold waves [49]. Correspondingly, the productivity of rubber tree in China is somewhat lower and usually 3-4 kg of dry rubber tree^-1^ yr^-1^. In such a growing condition it is spectacular to have this super-high-yielding PR107 tree (SY107) [18]. As calculated from the latex yield per tapping (Table 1), the SY107 produced 74.2 kg of dry rubber in 2006, the year when we conducted this study. This yield decreased markedly as compared to that (126 kg) in 2002 [18], but was still much higher than that of other rubber trees by average (5-6 kg of dry rubber tree^-1^ yr^-1^) growing in the same region and that of the two control trees (5.76 and 14.94 kg of dry rubber tree^-1^ yr^-1^, respectively) selected in this study at its proximity.

As the first step to investigate the mechanisms of high productivity in SY107, we compared four physiological parameters of the latex (the cytoplasm of rubber-producing laticifers) between SY107 and its controls. The four latex parameters, i.e. total solid content (TSC), sucrose, thiols and inorganic phosphorus (Pi) contents, revealed direct correlations with the production of certain rubber clones under certain conditions [33,50,51]. SY107 showed similar TSC and Pi contents to the controls, whereas its sucrose and thiols contents were significantly lower than the controls (Table 1). Sucrose is the raw material for laticiferous metabolism, particularly for rubber synthesis [52]. The sucrose content in the latex reflects the balance of sucrose loading and utilization in the laticifers. Sucrose utilization is always limited by the activity of a neutral/alkaline type of invertase, and thus constitutes one of the limiting factors for rubber production [52,53]. We hypothesize that the lower sucrose content in SY107 is due to more active sucrose utilization. This hypothesis was supported by a higher latex invertase activity (8.53 units/ml c-serum) in SY107 over the control trees (6.36 units/ml c-serum). In the laticifers, thiols are both protectors of the membrane systems and the activators of some key enzymes [33]. The lower latex thiols in SY107 may reflect higher consumption of thiols in SY107 to counteract the enhanced production of reactive oxygen species (ROS) under the conditions of a more active laticiferous metabolism [3]. Although the analysis of latex physiological parameters gave a hint to the mechanisms of high productivity in SY107, differences are not known in the molecular events that occur in the laticifers between SY107 and its controls.

The cDNA-AFLP technique allowed transcription changes to be surveyed with no prior assumption about which genes might be differentially expressed in the latex between SY107 and its controls. With two enzyme combinations selected, about 35000 cDNA fragments were visualized on the gels, and to our knowledge, this is one of the most extensive cDNA-AFLP analyses ever done in biological samples. A total of 352 genes were found to be differentially expressed, of which 215 showed significant homology to genes with known or partially known functions (Tables 2 and 3). The 215 genes belong to different functional categories, which indicates that SY107 and its controls differ in different physiological and biochemical pathways.

The up-regulation of a sucrose transporter *HbSUT3* (T3M10-2) suggests an enhanced sucrose loading to the laticifers of SY107. *HbSUT3* has been demonstrated to be actively involved in sucrose loading to laticifers and rubber productivity [14,15]. Moreover, two other sucrose transporters (*HbSUT5*, T13M1-2; *HbSUT2A*, A8M2-1) were also differentially expressed between SY107 and the control trees, further implicating the importance of the regulation of sucrose loading. In latex, both *HbSUT5* and *HbSUT2A* are less expressed than *HbSUT3* [14], but their roles in regulating sucrose loading deserve further studies. Sucrose catabolism in the latex is also an important factor affecting rubber productivity [52], and a neutral/alkaline invertase *HbNIN2* was identified as the key gene responsible for sucrose catabolism in the latex, and its expression was much higher in SY107 than the control trees (Tang et al., unpublished data). In this study, the cDNA-AFLP analysis failed to detect TDFs for *HbNIN2*. This may simply be due to our insufficient isolation of DE-TDFs since *in silico* restriction mapping indicates the suitability of *HbNIN2* mRNA for the cDNA-AFLP analysis. An INDETERMINATE DOMAIN transcription factor (TF) *AtIDD8* was reported in *Arabidopsis* to regulate sugar transport and metabolism by binding directly the promoters of a sucrose synthase gene (*SUS4*) [54]. It still needs to investigate whether in the laticifers some of the DE-TFs identified in this study (Tables 2 and 3) play roles in regulating the expression of *HbSUT3* and *HbNIN2* in similar ways to *AtIDD8*.

In the latex, rubber synthesis is thought to make better use of the energy and reducing power generated by carbohydrate fermentation rather than the Krebs cycle and oxidative phosphorylation [3], stressing the significance of glycolysis in rubber production. Phosphofructokinase (PFK) is one of the rate-limiting enzymes of glycolysis. In this study two PFK TDFs (T11M4-1; A1M9-2) were up-regulated, indicating an increased glycolysis in the laticifers of SY107. Also, the genes encoding a mannose-6-phosphate isomerase (A4M2-1) and an O-glycosyl compound hydrolase (A1M5-3) were identified, the former of which transforms mannose 6-phosphate into fructose 6-phosphate and then into glycolysis [55], and the latter hydrolyzes glycosidic linkages, such as those in cellulose and hemicellulose to release smaller sugars [56]. The up-regulation of these two genes suggests that SY107 has a higher capacity to mobilize other types of sugars as well as sucrose for subsequent glucidic metabolism. An alcohol dehydrogenase (T15M1-1) was observed up-regulated, which under the conditions of hypoxia facilitates the maintenance of glycolysis and the alleviation of cytosolic acidification [57], and favors latex regeneration [53]. In addition, a vacuolar-type inorganic pyrophosphatase (T15M3-4) was up-regulated, which functions in efficient turnover of pyrophosphate (PPi) produced during isoprene anabolism [3,58], and thus activates isoprene synthesis and glycolysis.

**Figure 4:**
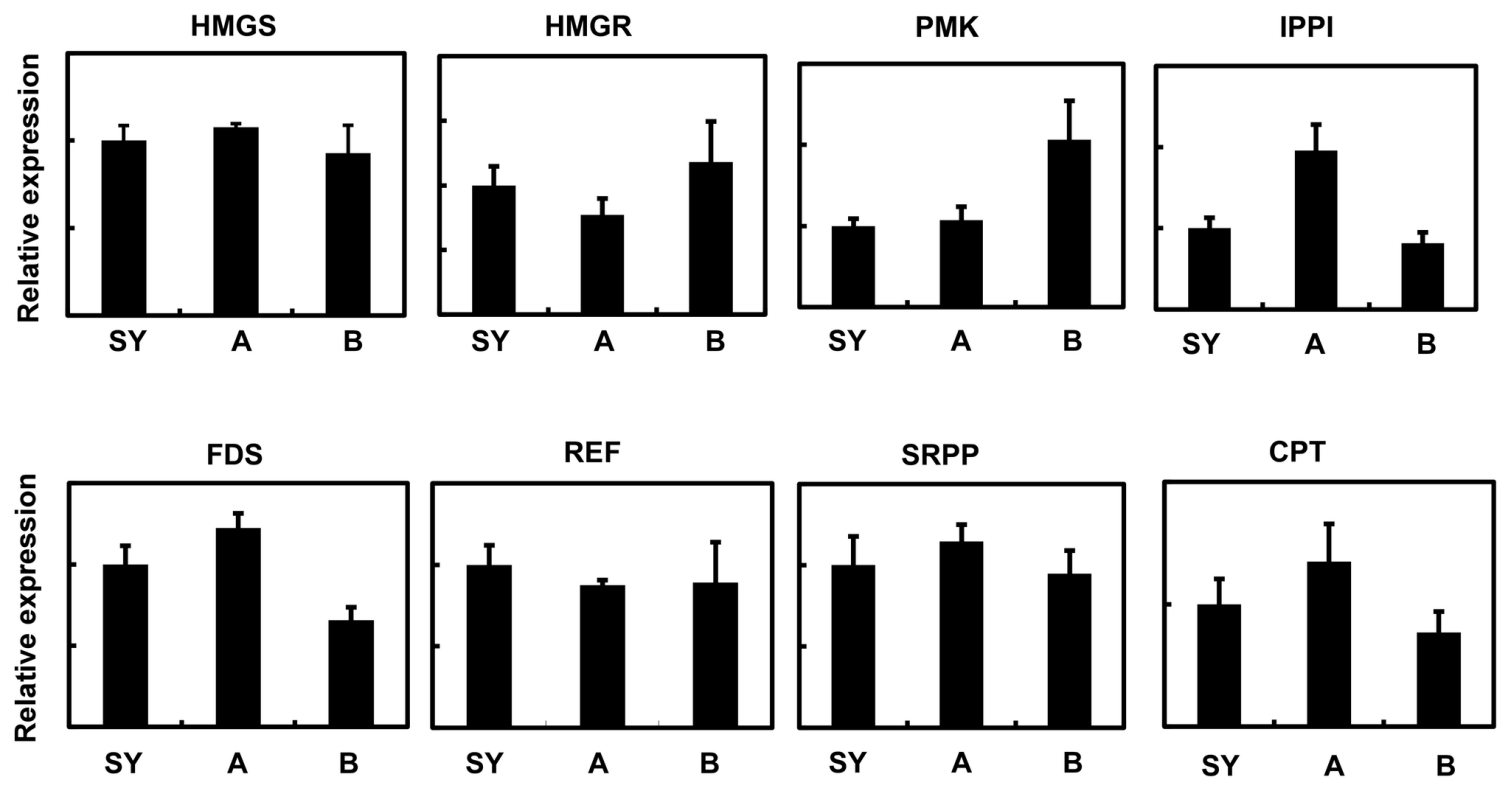
Expressional comparison of 8 rubber-biosynthesis-pathway genes between SY107 and its control trees by qRT-PCR. Abbreviations: HMGS, 3-hydroxy-3-methylglutaryl-coenzyme synthase; HMGR, 3-hydroxy-3-methylglutaryl -coenzyme reductase; PMK, phosphomevalonate kinase; IPPI, isopentenyl diphosphate isomerase; FDS, isopentenyl diphosphate synthase; REF, rubber elongation factor; SRPP, small rubber particle protein; CPT, cis-prenyl transferase. The values of relative expression are presented as the mean ± SE of three biological replicates. No genes showed significantly differential expression between SY107 and the two controls, as determined by *t* test.

The expressional regulation of the genes that are directly involved in the pathways running from acetyl-CoA to the *cis*-polyisoprene (rubber), and hence called rubber-biosynthesis pathway genes (RB-genes) is thought to be critical in affecting rubber productivity [6,59]. Several RB-genes, such as cis-prenyl transferase (CPT), small rubber particle protein (SRPP), rubber elongation factor (REF) and HMG-CoA reductase (HMGR), have been extensively studied, and the expression of REF in the latex was even found to correlate positively with the productivity of rubber clones (varieties) [11]. Although *in silico* restriction mapping confirmed the suitability of all known RB-genes for the cDNA-AFLP analysis, only one RB-gene (acetyl-CoA C-acetyltransferase, A2M8-1) was isolated in this study, and found down-regulated. Therefore, we set out to determine whether the failure to identify more RB-genes is due to insufficient DE-TDF isolation or just reflects their actual expression patterns between SY107 and other trees. As determined by qPCR, none of the eight RB-genes examined (HMG-CoA synthase, HMGR, phosphomevalonate kinase, IDP isomerase, FPP synthase, REF, SRPP and CPT) was significantly higher or lower expressed in SY107 than in the two controls (Figure 4). In another independent study, we compared the transcriptomes of three tissues (latex, bark and leaf) between two rubber clones (Reyan8-79 and Reyan7-33-97) using 454 GSFlx technology (Li et al. unpublished). Again, the differential expression of RB-genes in the latex of the two clones accounted little for the higher productivity of Reyan8-79 than Reyan7-33-97, further strengthening the idea that the SHY characteristics of SY107 has little correlation with the expression of RB-genes, but with the differential expression of the other genes involved in sucrose availability and catabolism, and other kinds of metabolisms within the latex cells.

Latex productivity of a rubber tree depends mainly on two factors: the duration of latex flow after tapping and the capability of latex regeneration between two consecutive tappings [3]. Latex regeneration is subjected to multiple biological processes, such as the regulation of transcription & translation, intracellular trafficking and signaling pathways, and water movement across the laticifers that is critical in controlling latex flow. In this study, the DE-genes involved in transcription and protein synthesis were most abundantly represented, the majority of which were up-regulated in SY107 (Tables 2 and 3), suggesting that during latex regeneration a much more enhanced gene expression and protein synthesis occurs in the latex of SY107. Also, latex regeneration requires the replenishment of large amounts of various organelles, especially the laticifer-specific ones (rubber particles, lutoids and Frey-Wyssling complexes), which adds up to 50-70% of the fresh latex volume [3]. The up-regulation of three important proteins, i.e. vacuolar protein sorting vps41 (A3M15-1), vesicle transport V-snare protein vti1a (T4M6-2) and ADP-ribosylation factor (T12M2-1), involved in intracellular trafficking, biogenesis and development of organells [60-62], agrees with the finding that SY107 has a much stronger capability of latex regeneration. In addition, signaling pathways, especially those of ethylene, jasmonate and wounding, are actively implicated in the regulation of latex regeneration [63]. In this study, nineteen genes encoding signaling components, including different types of kinases, receptors, G proteins and annex, were identified, most of which were up-regulated (Tables 2 and 3). The function of annex in signal transduction and amplification has been well described in plants [64], and here the up-regulation of an annex protein (T7M7-2), together with the up-regulation of many other signaling components, corroborates the idea that the stimulation of multiple signaling pathways contributes to the enhanced productivity in SY107. Moreover, the up-regulation of two aquaporins (A1M5-1; T11M8-2) was noticed in this study. One aquaporin (*HbPIP2;1*, T11M8-2) has been confirmed to have a role in regulating the water conduction between the laticifers and the inner liber tissues, and its expression is correlated positively with the ethylene stimulation of latex yield [16], suggesting that up-regulation of aquaporins contributes to an improved latex flow and thus the higher productivity in SY107.

Rubber harvesting, including the act of tapping and ethylene stimulation, produces osmotic and oxidative stresses [65]. The capability of a rubber tree, especially the rubber-producing cells - the laticifers - to tolerate, adapt and alleviate such stresses is essential for its sustainable rubber productivity. Among the 34 DE-genes in the category of defense and stress, 23 encode different families of heat-shock proteins (Hsps). The roles of Hsps in protein protection and cellular homeostasis have been made clear under cellular stress [66]. Remarkably, 21 of the 23 Hsps are down-regulated, whereas the other two Hsps, along with 11 other stress-related genes, are up-regulated (Tables 2 and 3). In plants, the over-expression of Hsp genes and other stress-related genes under stress conditions is usually realized at the cost of depressing other multiple metabolic genes, and thus affects plant normal growth and development [67,68]. The down-regulation of most DE-genes in the category of defense and stress and up-regulation of the vast majority of DE-genes on the whole suggest that the laticifers of SY107 are subjected to less stresses than other trees. This idea is further strengthened by the finding of the down-regulation of two Hsp70 proteins (A7M3-1 and A8M6-2), which are reported to be significantly over-expressed under stress conditions and can act as sensitive biomarkers of cell stress in humans [69], animals (snail species of the genus *Tegula*) [70] and plants (*Fucus serratus* and *Lemna* *minor* ) [71]. In *Arabidopsis* , most of the ROS metabolism-related genes are significantly regulated in stress conditions [72]. So, the result that no DE-TDFs for such kind of genes were isolated in this study further reveals a lower stress level in the latex of SY107. Together, these results suggest that SY107 has a better capability to lower stress levels in time and then enables to invest more effort into the metabolic pathways related to latex regeneration, and thus display a phenotype of higher productivity. Moreover, the up-regulation of multiple components of unbiquitin-mediated protein degradation and protein degradation-related signaling pathways was noticed (Table 2). A higher rate of protein turnover is essential for higher metabolism of plants [73,74], and it corroborates the extraordinarily active metabolism that occurred in the laticifers of SY107.

This is the first report of revealing markedly different yield levels of rubber trees through a global transcription comparison and analysis. The profiles of the large number of DE-genes identified in this work suggest that the SY107 tree has sufficient molecular basis for its super-high yielding, especially in the aspects of improved sucrose loading capability, RB-preferred sugar utilization, enhanced general metabolisms, and timely stress alleviation. However, it is noteworthy that RB-pathway genes seem to have little correlation with the high yielding phenotype. Next generation sequencing technologies, e.g. Solexa, SoLiD and 454, provide new tools to further exploring the mechanisms of super rubber productivity, since the powerfulness of these techniques in transcriptome profiling has been well documented [75]. Anyway, the results presented here provide a valuable guide for molecular rubber breeding aimed to enhance rubber production.

## Materials and Methods

### Ethics Statement

Our field studies and sampling have been approved and assisted by the owner of the rubber plantation, Yunan State Farms Group Co., LTD.

### Plant materials and growth conditions

Clone PR107 rubber trees used for cDNA-AFLP analysis have been cultivated in a plantation in Mengla County, Xishuangbanna Dai Autonomous Prefecture, Yunnan Province, China. These trees, including the super-high-yielding tree (SY107) and four average PR107 trees in its proximity, were planted in 1963 and opened in 1970. Since 2000 these trees have been tapped in a system of two half spiral cuts, every 4 days and with 4% Ethrel (an ethylene generator) stimulation (2×S/2DU. d4. ET4%. Pa. 20/y (15d). The four average PR107 trees were divided into two groups (107A and 107B) of two trees according to latex yields.

### Determination of latex yield, physiological parameters and invertase activity

The latex yields were determined from May to July 2006. In each month, the latex yields for three consecutive tappings after one round of Ethrel stimulation were measured. To assay the physiological parameters, an aliquot of 20 ml of latex per tree was collected 5 min after tapping in a 50-ml centrifuge tube placed in ice, and transported to the laboratory for immediate analysis. The parameters of total solid content (TSC), pH, thiols, inorganic phosphates, and sucrose contents were determined according to Eschbach et al. (1984) [33]. A fraction of fresh latex was centrifuged for 30 min at 4 °C and 45,000×*g*, and the clear middle phase (cytoplamic serum, c-serum) was collected for invertase activity analysis. The invertase activities were determined according to the procedure of Tupy (1969) [76] with modifications. Briefly, a 200-µl reaction mixture contained 50 µl c-serum, 0.1 mol/l of NaF and 0.06 mol/l of sucrose. The mixture was incubated at 30 °C for 1 h, and stopped by adding 2 volumes of alcohol and boiling for 5 min. Then, distilled water was added to adjust the mixture to a final volume of 1.5 ml, and centrifuged to collect the clear solution for determining the reducing sugars by the method of Miller (1959) [77]. One unit of invertase activity is defined as the amount of enzyme that which produces 1.0 µmoles of reducing sugars per minute at 30 °C. The number of units given in the results is expressed per ml of c-serum.

### RNA extraction, cDNA synthesis and cDNA-AFLP analysis

During the collection of latex for determining physiological parameters, an aliquot of 5 ml of latex per tree was sampled concomitantly for RNA extraction, cDNA synthesis and cDNA-AFLP analysis. Experimental manipulations were conducted according to the methods previously described [30,78]. Total RNA concentration was determined spectrophotometrically and adjusted to a final concentration of 1 µg μl^-1^. Poly(A)^+^ RNA was enriched from a 50-µg aliquot of total RNA for each sample using polyTract® mRNA isolation systems III (Promega, Madison, WI). The enriched poly (A)^+^ RNA was reverse-transcribed with oligo[dT] 25V (V=A, C or G) primer into first strand cDNA by using the RevertAidTM First Strand cDNA synthesis kit (Fermentas, Lithuania). The first strand cDNA was then transformed into double-stranded (ds) cDNA using DNA polymerase I (Takara Bio, Dalian) and RNase H (Fermentas, Lithuania) according to the manufacturers’ manuals. The resulting ds-cDNA was extracted with phenol: chloroform: isoamyl alcohol (25:24:1), and then ethanol precipitated with linear polyacrylamide as the carrier [79]. The ds-cDNA precipitate was dissolved in double distilled water (ddH_2_O) for template preparation.

The ds-cDNA was digested by one of the two restriction enzyme pairs selected, *Apo*I/*Mse*I and *Taq*I/*Mse*I, and used for the preparation of pre-amplification templates. All 384 possible selective primer combinations for the two restriction enzyme systems (Table S1) were employed to perform transcriptional profiling. The details of cDNA-AFLP analysis were previously described [30].

### Isolation and sequencing of transcript-derived fragments (TDFs)

All bands longer than 70 bp were compared in the three samples (SY107, 107A and 107B), and those differentially expressed (DE) TDFs, varying in length from 70-800 bp, were exercised from the gels, re-amplified, and sequenced. The DE-TDFs were classified into two categories with the expression level in SY107 compared to that in the two controls (107A and 107B): up-regulated and down-regulated, with a criterion of ≥2-fold difference in band intensities. The bands corresponding to differentially expressed TDFs between SY107 and the controls (107A and 107B) were excised from the PAGE gel. The gel bands were soaked in 20 µl of sterile 10 mM Tris-HCl buffer containing 1 mM EDTA, pH 8.0, initially at 95 °C for 30 min and then hydrated overnight at 4 °C, and followed by centrifugation at 10,000*g* for 2 min to collect the supernatant. An aliquot of 2 µl supernatant was included in a 40 µl mixture for re-amplification, using the corresponding selective primers (Table S1) and the same conditions that were in pre-amplification. The amplified fragments were fractionated on a 1.5% agarose gel. The band with the target size was isolated and then sequenced using ABI PRISMTM 3730 DNA Sequencer in Shanghai GeneCore BioTechnologies Co., Ltd (Shanghai, China).

### Sequence analysis

To analyze a large set of TDF data, we built a local stand-alone EST analysis platform. First, PHRAP package [http://www.phrap.org/phredphrapconsed.html] was used to pre-process the TDF data. ABI files derived from the ABI PRISMTM 3730 DNA Sequencer were converted into text files using PHRED with default parameters [80,81]. Vector sequences were then eliminated by CROSS_MATCH with the parameters of -minmatch 10 -miniscore 20. All TDFs, in the format of FASTA, were clustered using CAP3 with the parameters of -o 40 –p 90 [http://genome.cs.mtu.edu/cap/cap3.html], with 21 as the overlap parameter [82]. BLAST analysis was conducted to the filtered unique TDFs with stand-alone BLAST [ftp://ftp.ncbi.nlm.nih.gov/blast/executables/release/] package using BLASTX as the engine, NR [ftp://ftp.ncbi.nlm.nih.gov/blast/db/] as the database, and E-value of 1e-5 as the cutoff point for acceptance of similar functions. Sequences were manually assigned to functional categories as described by Bevan et al. [83], and assisted by the relevant scientific literature and the information reported for each sequence by the Gene Ontology consortium, when available, or reported by the Swiss-Prot [http://expasy.org/sprot/], KEGG [http://www.genome.jp/kegg/] [84] and TAIR databases [http://www.arabidopsis.org/].

### QPCR Analysis

For qPCR, first-strand cDNA was synthesized using 2.5 µg of DNase I-treated total latex RNA as described in Li et al. [85]. The PCR reaction was performed in a LightCycler 2.0 system (Roche Diagnostics, Germany). Oligonucleotide primer pairs (forward and reverse) for 18 randomly selected target genes (Table S2) and eight rubber biosynthesis pathway genes (Table S3) were designed according to the known DNA sequences from TDFs. The *UBC2b* gene, determined to be the most suitable internal control for individual rubber trees [85], was used as the reference gene. The relative abundance of transcripts was calculated according to the instructions of the LightCycler Relative Quantification Software 4.05: Expression =E^Ct(UBC2b)-Ct(target gene)^. All amplified fragments were cloned, and sequenced for target confirmation. For each target gene, three independent qPCR analyses were performed. The experimental details were described previously [85].

### Statistical Analysis

The Student’s *t* test was used to compare the expression of the selected genes or TDFs in latex between SY107 and its controls using the algorithm embedded into Microsoft Excel 2007. The term “significant” is used in the text only when the change in question has been confirmed to be significant (P < 0.05).

## Supporting Information

Table S1 **Adaptors and primers used for cDNA-AFLP analysis.**
(DOC)

Table S2 **Primers used in quantitative real-time PCR (qRT-PCR) for validation of cDNA-AFLP results.**
(DOC)

Table S3 **Primers used in qRT-PCR for expressional comparison of 8 rubber-biosynthesis-pathway genes between SY107 and its control trees.**
(DOC)
