# Supplementary material for: Comparative Analysis of Latex Transcriptome Reveals Putative Molecular Mechanisms Underlying Super Productivity of Hevea brasiliensis
Source: PLoS One. 2013 Sep 16;8(9):e75307. doi: 10.1371/journal.pone.0075307 (PMC3774812; doi:10.1371/journal.pone.0075307)
Supplement: Table S1 — Adaptors and primers used for cDNA-AFLP analysis. (DOC) [file pone.0075307.s001.doc]

**Table S1**

| Usage | Primer Name | Sequence (5’→3’) |
| --- | --- | --- |
| *Apo*I adaptor top strand | *Apo*I-TS | CTCGTAGACTGCGTACC |
| *Apo*I adaptor bottom strand | *Apo*I-BS | AATTGGTACGCAGTCTAC |
| *Taq*I adaptor top strand | *Taq*I-TS | GACGATGAGTCCTGAC |
| *Taq*I adaptor bottom strand | *Taq*I-BS | CGGTCAGGACTCAT |
| *Mse*I adaptor top strand | *Mse*I-TS | GACGATGAGTCCTGAG |
| *Mse*I adaptor bottom strand | *Mse*I-BS | TACTCAGGACTCAT |
| *Apo*I pre-amplification primer | *Apo*I-PP | CTCGTAGACTGCGTACCAATT |
| *Taq*I preamplification primer | *Taq*I-PP | GACGATGAGTCCTGACCGA |
| *Mse*I preamplification primer | *Mse*I-PP | GACGATGAGTCCTGAGTAA |
| *Apo*I selective primers | A1 | GACTGCGTACCAATTTA |
|  | A2 | GACTGCGTACCAATTTT |
|  | A3 | GACTGCGTACCAATTTC |
|  | A4 | GACTGCGTACCAATTTG |
|  | A5 | GACTGCGTACCAATTCA |
|  | A6 | GACTGCGTACCAATTCT |
|  | A7 | GACTGCGTACCAATTCC |
|  | A8 | GACTGCGTACCAATTCG |
| *Mse*I selective primers | M1 | GATGAGTCCTGAGTAAAA |
|  | M2 | GATGAGTCCTGAGTAAAT |
|  | M3 | GATGAGTCCTGAGTAAAC |
|  | M4 | GATGAGTCCTGAGTAAAG |
|  | M5 | GATGAGTCCTGAGTAATA |
|  | M6 | GATGAGTCCTGAGTAATT |
|  | M7 | GATGAGTCCTGAGTAATC |
|  | M8 | GATGAGTCCTGAGTAATG |
|  | M9 | GATGAGTCCTGAGTAACA |
|  | M10 | GATGAGTCCTGAGTAACT |
|  | M11 | GATGAGTCCTGAGTAACC |
|  | M12 | GATGAGTCCTGAGTAACG |
|  | M13 | GATGAGTCCTGAGTAAGA |
|  | M14 | GATGAGTCCTGAGTAAGT |
|  | M15 | GATGAGTCCTGAGTAAGC |
|  | M16 | GATGAGTCCTGAGTAAGG |
| *Taq*I selective primer | T1 | GATGAGTCCTGACCGAAA |
|  | T2 | GATGAGTCCTGACCGAAT |
|  | T3 | GATGAGTCCTGACCGAAC |
|  | T4 | GATGAGTCCTGACCGAAG |
|  | T5 | GATGAGTCCTGACCGATA |
|  | T6 | GATGAGTCCTGACCGATT |
|  | T7 | GATGAGTCCTGACCGATC |
|  | T8 | GATGAGTCCTGACCGATG |
|  | T9 | GATGAGTCCTGACCGACA |
|  | T10 | GATGAGTCCTGACCGACT |
|  | T11 | GATGAGTCCTGACCGACC |
|  | T12 | GATGAGTCCTGACCGACG |
|  | T13 | GATGAGTCCTGACCGAGA |
|  | T14 | GATGAGTCCTGACCGAGT |
|  | T15 | GATGAGTCCTGACCGAGC |
|  | T16 | GATGAGTCCTGACCGAGG |
