# Supplementary material for: Comparative Analysis of Latex Transcriptome Reveals Putative Molecular Mechanisms Underlying Super Productivity of Hevea brasiliensis
Source: PLoS One. 2013 Sep 16;8(9):e75307. doi: 10.1371/journal.pone.0075307 (PMC3774812; doi:10.1371/journal.pone.0075307)
Supplement: Table S2 — Primers used in quantitative real-time PCR (qRT-PCR) for validation of cDNA-AFLP results. (DOC) [file pone.0075307.s002.doc]

**Table S2**

| TDF | Gene Name | Primer type | Sequence (5’→3’) |
| --- | --- | --- | --- |
| T12M1-1 | USP-like protein | forward | CACCGCTTGATTTTGGTTTCT |
|  |  | reverse | TGAGTGCGGACATGCTAGTGA |
| T8M3-3 | dehydrin | forward | TTTTTTCCCCCTAAGTTTTCTGT |
|  |  | reverse | TCTCTTCCTCTTTCTTCCCCAT |
| T7M14-1 | integral membrane HRF1 family protein | forward | GCACCAAGTCCTCCTCTAATAAG |
|  |  | reverse | CCTCCAACAAATCCTCAGCCTA |
| T15M1-1 | alcohol dehydrogenase | forward | AGCTTTCATTATGCCTTGTGGT |
|  |  | reverse | TGCGGAGGAATAGCCTTGTT |
| T2M12-2 | short-chain dehydrogenase/reductase SDR | forward | GAGCCTTGCCAATTCCATCA |
|  |  | reverse | AGGTGGGTATGGGTCATGTTCT |
| T9M8-1 | cytochrome c oxidase subunit Vc family protein / COX5C family protein | forward | GCTCTGTAGGCTCACCCTGTCT |
|  |  | reverse | TTGTGGAAAATGAATCAATGGA |
| T16M8-1 | ketoacyl-ACP Reductase (KAR) | forward | TTCTCATCAACAATGTATCTCGGG |
|  |  | reverse | AGATTGAGGCTTATGGTGGTGA |
| T11M3-2 | trehalose-6-phosphate synthase | forward | AACACTTCGGCCCTCGG |
|  |  | reverse | GCTTGTGGCTAAACGCCTACT |
| T15M1-3 | eukaryotic translation initiation factor | forward | TCACGGAAAGGTTTGATGGAG |
|  |  | reverse | TATTTATAGTTGCCTGGCGAGAT |
| T13M2-3 | ubiquitin fusion protein | forward | GCTGAGGCTTAGGGGAGG |
|  |  | reverse | ATGACCGCATTTCTTCTTTCG |
| T15M3-2 | AP2/ERF domain-containing transcription factor | forward | TGATGAAGCTGCAAGGCTTATG |
|  |  | reverse | TTGAGGGCGATGATTGTGAT |
| T7M11-1 | heat-shock protein | forward | TTTGCTAACACTCGCATGGACT |
|  |  | reverse | TTGTCTTCCTTCTCCACATTCC |
| A5M9-2 | heat-shock protein | forward | TAGAGGTTGATGAAGGGAGGGT |
|  |  | reverse | TTAGGCACAGTGACAACAAGCA |
| T13M13-1 | adrenodoxin | forward | CTGGTGGTGTTATTCAGTTGTTT |
|  |  | reverse | CTTTTCCTCTCCATCCTTATCC |
| T13M1-2 | sucrose transporter 5 | forward | CCACATTGGCTATTTAGGTCAT |
|  |  | reverse | CCAAGTCCCAAAGGTTCAATC |
| T9M13-3 | CCR4-associated factor | forward | GGAGGTGGAAAGAGTTGGTGTC |
|  |  | reverse | CCGCTGAAAAAGTCGTCTCTC |
| T10M4-3 | protein kinase 2 | forward | TTAGAGCCATAACCACATCCGA |
|  |  | reverse | GATTGTACCAAGCTCTCGCCA |
| A4M1-2 | heat-shock protein | forward | GAGTGCTTCAAATCAGCGGTG |
|  |  | reverse | GCTCCACACGATGCCAAGTAT |
