# Supplementary material for: Comparative Analysis of Latex Transcriptome Reveals Putative Molecular Mechanisms Underlying Super Productivity of Hevea brasiliensis
Source: PLoS One. 2013 Sep 16;8(9):e75307. doi: 10.1371/journal.pone.0075307 (PMC3774812; doi:10.1371/journal.pone.0075307)
Supplement: Table S3 — Primers used in qRT-PCR for expressional comparison of 8 rubber-biosynthesis-pathway genes between SY107 and its control trees. (DOC) [file pone.0075307.s003.doc]

**Table S3**

| Gene name | GenBank acc. no. | Sequence (5’→3’) |
| --- | --- | --- |
| HMGS | AF396829 | CAAGCCCCTTTATGATGCGA; AGAGAACAGTGTCACCCGCTTG |
| HMGR | AY706757 | GCATCTCAGTCTGCTTGTCTCA; TCTTTGCTGGATCTGTTGTACTT |
| PMK | AF429385 | GCAGGTGTTCCGATAGAGCC;  AGAGTCCCCTAAGGTAACAGCG |
| IPPI | AB294696 | CGCTTCCCTCCTCTGCTTCT;  GGTGGAAGTAGCGGTGATTGC |
| FDS | AB294712 | CAATGGGTTGAGCGGATGTT;  TGACCACGTCGTGTATGAGAGC |
| REF | AY712939 | AGCCTCCATCAGCGTTTTCAGA;  GCCCTTGTTGGTTGTCTTCGTC |
| SRPP | HQ640231 | TCTTTTTTCAAACTTCAGCGAC;  ATCTACAGCATAAACTCCAGCC |
| CPT | AB294716 | GTGAGCCAGTCAAGACCGCA;  TTCCAGTTGCATTTGCCTCC |
